# Promising Mechanical, Thermal, and Ballistic Properties of Novel Epoxy Composites Reinforced with *Cyperus malaccensis* Sedge Fiber

**DOI:** 10.3390/polym12081776

**Published:** 2020-08-08

**Authors:** Lucas de Mendonça Neuba, Raí Felipe Pereira Junio, Matheus Pereira Ribeiro, Andressa Teixeira Souza, Eduardo de Sousa Lima, Fábio da Costa Garcia Filho, André Ben-Hur da Silva Figueiredo, Fábio de Oliveira Braga, Afonso Rangel Garcez de Azevedo, Sergio Neves Monteiro

**Affiliations:** 1 Department of Materials Science, Military Institute of Engineering-IME, Rio de Janeiro 22290270, Brazil; lucasmneuba@gmail.com (L.d.M.N.); raivsjfelipe@hotmail.com (R.F.P.J.); m.pereiraribeiro@hotmail.com (M.P.R.); andressa.t.souza@gmail.com (A.T.S.); sousalima@ime.eb.br (E.d.S.L.); abenhur@ime.eb.br (A.B.-H.d.S.F.); snevesmonteiro@gmail.com (S.N.M.); 2 Department of Mechanical and Aerospace Engineering, University of California San Diego—UCSD, La Jolla, CA 92093, USA; fdacostagarciafilho@eng.ucsd.edu; 3 Department of Civil Engineering, Federal Fluminense University—UFF, Niterói 24210240, Brazil; 4 Department of Agricultural and Environmental Engineering, Federal Fluminense University—UFF, Niterói 24210240, Brazil; afonso.garcez91@gmail.com

**Keywords:** natural fiber composite, *Cyperus malaccensis*, sedge, mechanical properties, thermal analysis, ballistic armor

## Abstract

Composites reinforced with natural lignocellulosic fibers (NLFs) are gaining relevance as the worldwide demand for renewable and sustainable materials increases. To develop novel natural composites with satisfactory properties, less common NLFs should also be investigated. Among these, the *Cyperus malaccensis* (CM), a type of sedge fiber, is already used in simple items like ropes, furniture, and paper, but has not yet been investigated as composite reinforcement for possible engineering applications. Therefore, the present work evaluated for the first time the properties of novel epoxy composites incorporated with 10, 20, and 30 vol.% of CM sedge fibers. Tensile, Izod-impact, and ballistic impact tests were performed, as well as Fourier transform infrared (FT-IR) spectroscopy and thermal analysis of the composites. Results disclosed a decrease (−55%) in tensile strengths as compared to the neat epoxy. However, the elastic modulus of the 30 vol.% sedge fiber composite increased (+127%). The total strain and absorbed ballistic energy did not show significant variation. The Izod impact energy of the 30 vol.% composite was found to be 181% higher than the values obtained for the neat epoxy as a control sample. An increase in both stiffness and toughness characterized a reinforcement effect of the sedge fiber. The thermal analysis revealed a slight decrease (−15%) in the degradation temperature of the CM sedge fiber composites compared to the neat epoxy. The glass-transition temperatures were determined to be in the range of 67 to 81 °C.

## 1. Introduction

Synthetic fibers experienced exponential growth after the Second World War and became a successful class of engineering materials, mainly as reinforcement of polymer matrix composites. They have been applied in several technological fields of human interest, from surgical prostheses to aerospace components [1,2,3]. However, at the end of the last century, concerns on the widespread pollution caused by non-degradable materials, such as common plastics, as well as climate changes resulting from carbon dioxide emissions, motivated a tendency to replace synthetic by natural fibers in composite materials. These fibers, naturally produced by living organisms, such as animals [4] and plants [5], consume less energy to be processed and rapidly degrade in natural environments, reducing disposal problems and contributing to the sustainability of the projects.

Cellulose-rich natural lignocellulosic fibers (NLFs) have been gaining considerable interest as possible substitutes for the reinforcement phase of polymer composites [5,6,7,8,9,10,11,12,13,14,15,16], including totally biodegradable biocomposites [17]. Indeed, as compared to synthetic fibers, the NLFs display better characteristics, such as lower density and cost, as well as superior specific strength and elastic modulus [18,19]. Such characteristics are motivating applications in automotive, packaging, civil construction, and aerospace industries associated with NLF composites [20,21,22,23,24,25,26]. In particular, these composites are being considered for ballistic armors [27,28,29,30]. More recently, several researchers have been investigating the NLF waste as a source of nanocrystalline cellulose (NCC) [31,32,33,34,35,36], a strong nanomaterial, which can be used to produce several important products, such as nanocomposites, ion exchange membranes, films for electronics devices, and others [36].

Despite all the advantages, NLFs present some drawbacks associated with their polar incompatibility towards a non-polar nature of polymeric matrices. In this case, poor interfacial adhesion between the reinforcement and matrix phases is created, impairing the mechanical properties [6,7,8,9,10,11,12,13,14]. Another major limitation often encountered is thermal degradation. The decomposition reaction of the fiber components (cellulose, hemicellulose, and lignin) mainly occurs at low temperatures, around 200 °C, and imposes limitations to processing and application for the NLF-reinforced polymeric-matrix composites [37]. In the case of less known NLFs, it is important to study the thermal behavior of related polymer composites, in order to assure better processing and properties [38,39]. Among the less known NLFs, the several types of sedge only recently begun to be investigated [40,41,42,43,44,45,46,47] but have still not been cited in the aforementioned specialized reviews dedicated to technical properties of NLF polymer composites.

Babu et al. [40] evaluated polyester composites reinforced with *Cyperus pangorei* fibers treated with benzoyl chloride. They studied the tribological properties of the composites aiming the application as vehicle brakes. Babu et al. [41] investigated the mechanical and thermal properties of the *Cyperus esculentus* fiber-reinforced polypropylene composites, with and without mercerization. The mercerized specimens presented the best properties, as well as the 30 vol.% fiber specimens. They obtained values as high as ~70 MPa of tensile strength, ~60 MPa of flexural strength, and 30 J/m impact strength. Rajini et al. [42] studied the tribological properties of *Cyperus pangorei* fiber-reinforced polyester composites, such as hardness, friction coefficient, and surface roughness. Kalimuthu et al. [43] examined the mechanical properties and morphology of *Cyperus pangorei* fiber-reinforced polyester composites. They evaluated several fiber lengths and percentages of fibers. The best performance was observed for the 40 mm length fiber and 40 vol.% fraction of fibers. The tensile strength was found as 57.7 MPa, and the flexural strength as 90.5 MPa. Vijay and Singaravelu [44] investigated the influence of the sandwiching stacking sequence of *Cyperus pangorei* fiber layers in the mechanical properties of laminated composites. They produced the specimens by stacking the *C. pangorei* layers within external glass fabric layers either with kenaf, sisal, or jute core layers. The best results were obtained by the kenaf-cored laminates, which presented 51.5 MPa as tensile strength, 384.3 MPa as flexural strength, and 8.17 J as impact strength. Mayandi et al. [45] performed a thorough characterization (mechanical, thermal, chemical, and structural) of the *Cyperus Pangorei* fibers. The values 196 ± 56 MPa and 11.6 ± 2.6 GPa were obtained as tensile strength and elastic modulus for the fibers, respectively. They also found out that the fibers were semicrystalline (~41% crystallinity obtained by XRD) and were stable until 221 °C. The general characteristics of these fibers, according to the authors, make them promising to composite reinforcement. Govindasamy et al. [46] evaluated the characteristics of the *Cyperus malaccensis* (CM) fibers for papermaking purposes. Benazir et al. [47] studied the chemical composition, structure, and some mechanical properties of mat sedge *Cyperus pangorei* yarns. They found out that the fibers were composed mainly of alpha-cellulose (~42%), hemicellulose (~42%), lignin (~13%), and waxes (~2%).

In particular, the CM sedge fiber has not yet been investigated for possible composite reinforcement but only for papermaking [46] or agricultural purpose [48]. Therefore, the objective of this work was for the first time to investigate the mechanical and thermal behavior of the CM sedge fiber-reinforced composites. Tensile, Izod-impact, and ballistic impact tests were performed, as well as Fourier transform infrared (FT-IR) and thermal analysis of the fibers and composites.

## 2. Materials and Methods

### 2.1. Materials

**Figure 1:**
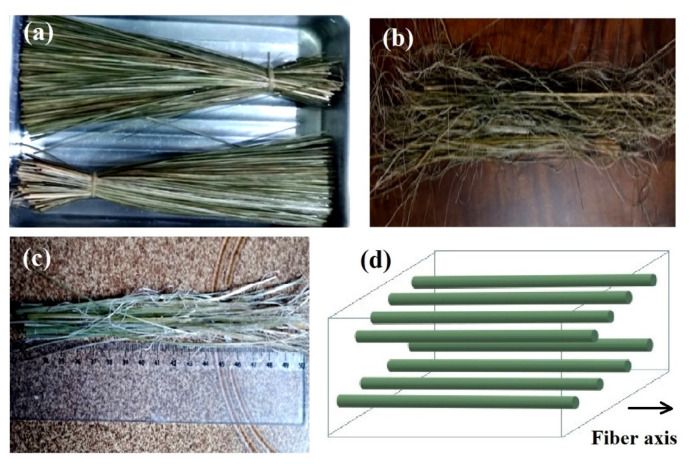
The extraction process of the fibers: (**a**) *Cyperus malaccensis* (CM) sedge immersed in water for a period of 24 h; (**b**) shredded fibers; (**c**) fibers already cut 150 mm in length; (**d**) schematic diagram of the aligned arrangement of the fibers in the composite.

The CM sedge fibers were extracted from a plant known in China as “mat grass” and in Brazil as *junco sete-ilhas* (in a free translation “seven-islands sedge”). These CM sedges, found in the Brazilian southeast region of the *Vale do Ribeira*, are illustrated in Figure 1. A CM mat was acquired from the company Artevale, São Paulo, Brazil.

A commercial epoxy resin, diglycidyl ether of bisphenol A (DGEBA)-type, hardened with triethylene tetramine (TETA) in a 13 phr stoichiometric ratio, was used as a polymeric matrix. It was fabricated by the company Dow Chemical, São Paulo, Brazil, and distributed by the company Resinpoxy Ltd.a, Rio de Janeiro, Brazil.

The extracted fibers were manually cleaned, immersed in water for 24 h, Figure 1a, shredded, Figure 1b, cut to a 150 mm length, Figure 1c, and then dried at 70 °C for 24 h. These fibers were not subjected to any chemical treatment. The immersion technique was adopted for providing greater flexibility and thus contributing to making the defibration process easier. Cutting tools helped in the process, assisting the removal of continuous fibers without sudden rupture, making it possible to obtain fibers with a larger length and quantity.

### 2.2. Composite Processing

To produce the composite plates, a metal mold with rectangular dimensions of 150 × 120 × 12 mm was used. The aligned fibers were carefully accommodated inside the mold’s cavity, schematically illustrated in Figure 1d, and the resin-hardener mix was poured into the mold in a previously calculated fiber/resin ratio. For these calculations, the fiber was measured by a geometric linear density method, which consists of precision-weighting about 100 fibers and measuring their diameter and length using an optical microscope. The calculated fiber density values were obtained by dividing the weight by the calculated volume, and then the mean value of 0.46 g/cm^3^ was obtained. For the epoxy resin, the density value of 1.11 g/cm^3^ was taken from the literature [49]. Specimens with 10, 20, and 30 vol.% fibers were produced. Finally, the mixture was kept under pressure of 5 tons for 24 h to assist the curing process. The final cure was performed at 60 °C for one hour.

### 2.3. FT-IR Analysis

Fourier transform infrared (FT-IR) spectroscopy was performed in a model IR Prestige 21-FTIR Shimadzu equipment (Tokyo, Japan).

### 2.4. Impact Tests

For the Izod impact tests, 10 specimens for each group were cut from the composite plates, following the dimensions of the ASTM D256 standard [50] (60.25 × 12.7 × 10 mm). A 45 ± 1° and 2.54 mm-deep V-notch was produced in each specimen, using a manual Pantec Iz/Ch-50 single-tooth carver. The impact tests were carried out in a model XC-50 Pantec machine, using a 22J hammer.

### 2.5. Tensile Tests

For the tensile tests, 6 specimens for each group were cut from the composite plates, following the dimensions of the ASTM D3039 standard [51]—150 × 15 × 2 mm and gauge length of 90 mm. The tests were conducted in an EMIC 23 equipment (Instron, São José dos Pinhais, Brazil), with a load capacity of 20 kN and a crosshead speed of 2 mm/min.

### 2.6. Ballistic Tests

Composite plates were prepared for the ballistic tests, one for each condition, with the dimensions of 15 × 12 × 19 cm^3^, comprising of 5 test-shots for each plate. A Gunpower SSS pressure air rifle (Ashford, UK) (4000 psi) was used to perform the test-shots, with 22 mm commercial ammunition (estimated mass of 3.3 g). A model MK3 Air Chrony gun chronograph (Nové Město, Czech Republic), with a precision of 0.15 m/s, was used for the impact velocity measurements. A model Pal ProChrono gun chronograph (Competition Eletronics, Rockford, IL, USA), with a precision of 0.31 m/s, was used for the residual velocity measurements. The former was positioned 10 cm before the target to measure the impact velocity, and the latter was placed 10 cm after the target to measure the residual velocity. The specimens were firmly positioned 5 m away from the rifle, and the bullet trajectory was perpendicular to the target plate.

As mentioned before, the projectile’s velocity was measured immediately before (*V_i_*) and after (*V_r_*) the impact. The kinetic energy variation was related to the energy absorbed by the target (E_abs_) and used for comparison between the tested materials [52].

(1) $$E_{abs}=\frac{m\left(V_{i}^{2}-V_{R}^{2}\right)}{2}$$

where: *m* = mass of the projectile.

Another calculation was performed for the limit velocity (*V_L_*), which is estimative of the velocity that the target would be able to stop the projectile, once *V_r_* is considered zero.

(2) $$V_{L}=\sqrt{\frac{2E_{abs}}{m}}$$

The results of the mechanical and ballistic tests were statistically treated by analysis of variance (ANOVA) to verify if there were significant differences between the averages, with a 95% confidence level. The mean values were then compared using Tukey’s test, also called honestly significant difference (HSD), calculated as follows.

(3) $$\mathrm{HSD}=q\sqrt{\frac{EMS}{r}}$$

where: *q* = HSD constant tabulated for 5% significance; *EMS* = error mean square of the ANOVA; *r* = number of repetitions for each treatment [53].

### 2.7. Thermal Analysis

Thermal analyses of the composites were performed by two methods: thermogravimetry (TGA) and differential scanning calorimetry (DSC). The materials were comminuted and placed in a platinum crucible. A model DTG-60H Shimadzu equipment (Tokyo, Japan) was used, with a heating rate of 10 °C/min, starting at 30 up to 600 °C, on a nitrogen atmosphere, with a gas flow of 50 mL/min.

### 2.8. Materials

Microscopic analyses of the fibers were performed by scanning electron microscopy (SEM) in a model Quanta FEG 250 Fei microscope (Thermofisher scientific, Hillsboro, OR, USA), operating with secondary electrons between 5 and 15 kV.

## 3. Results and Discussion

**Figure 2:**
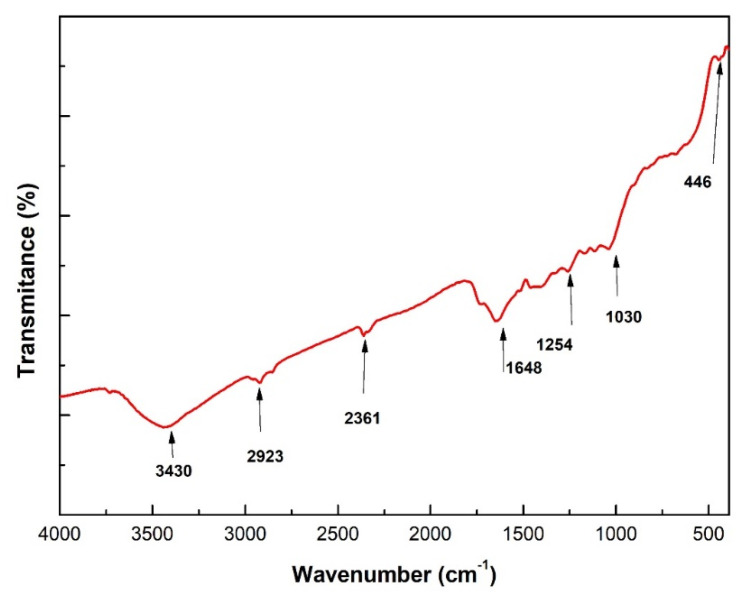
Fourier transform infrared (FT-IR) spectrum of the CM sedge fiber.

The Fourier transform infrared (FT-IR) spectrum of the CM sedge fiber is shown in Figure 2. In this spectrum, an absorption band appears at 3430 cm^−1^, which is attributed to the axial vibration of hydroxyl groups (O-H) of the cellulose [54]. The band found at 2923 cm^−1^ refers to the vibration of CH_2_ and CH_3_ molecules of the organic structure of NLFs. The relatively small amplitude of this band in the CM sedge fiber might justify a low interaction with the polymer matrix during the eventual manufacture of composites [54]. The band at 2361 cm^−1^ is assigned to the bond between organic molecules. The band at 1648 cm^−1^ is related to C=O aromatic groups, and the band at 1254 cm^−1^ to the C–O and C–C bonds. The bands at 1030 and 446 cm^−1^ are attributed to C–O and C–C deformation of the respective covalent bonds.

**Figure 3:**
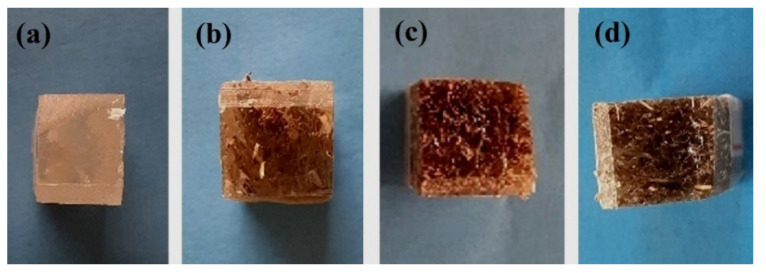
Fractured specimens after Izod impact test: (**a**) neat epoxy resin; (**b**) 10 vol.% of CM fibers; (**c**) 20 vol.% of CM fibers; (**d**) 30 vol.% of CM fibers.

Table 1 presents the results for all Izod impact tests for the different composite materials. They showed a low energy absorption capacity when compared to other different composites reinforced with natural lignocellulosic fibers (NLFs) [55,56]. The tested specimens were completely fractured, as expected, which is required for the validity of the tests, as shown in the macrographs in Figure 3.

**Figure 4:**
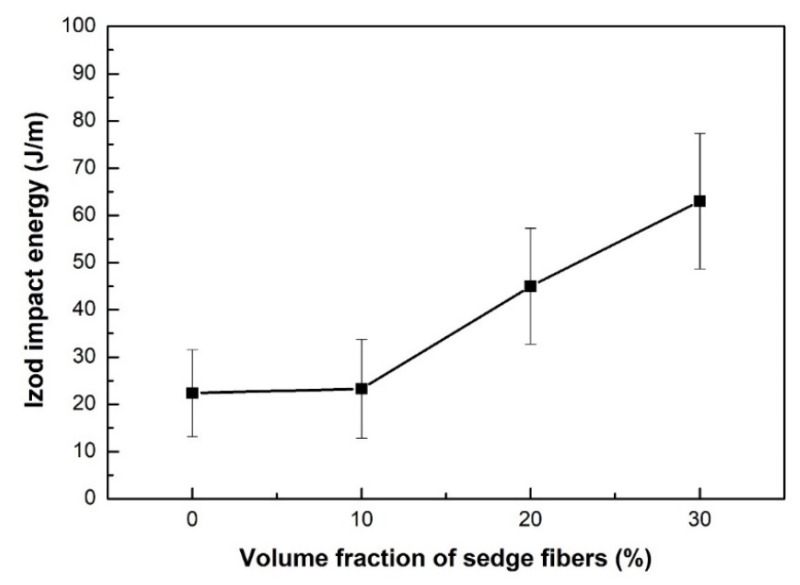
Izod impact energy as a function of the fiber fraction for the neat epoxy resin and CM fiber-reinforced composites.

The results in Table 1 can be better visualized by the graph in Figure 4. A tendency of increasing impact energy with increasing fiber fraction was observed, as already observed for other NFL polymer composites [55,56]. The effect of incorporating 30 vol.% fibers as reinforcement in composites was remarkable, producing a 181% increase when compared to the value obtained for the neat epoxy resin.

ANOVA was conducted to confirm if there is a significant difference between the Izod impact energy average results. According to Table 2, the hypothesis that the averages are equal was rejected, with a level of significance of 5%, since the calculated F (statistical parameter of ANOVA) was higher than the critical F_c_ (tabulated). Therefore, it was confirmed that there was indeed a significant difference between the impact energy for the different materials.

Table 3 shows the results of the Tukey’s honestly significant difference (HSD) test. The calculated HSD was 16.32 J/m, and thus the differences above the HSD were considered significant. These values are marked in bold in Table 3 and showed that the impact strength of the 30 vol.% sedge fiber composites was better than all other tested specimens.

The impact test results were now compared to the results for the other fibers of the *Cyperus* family, investigated as possible reinforcement to polymers, as presented in Table 4 [41,43]. The composites of the present work displayed better impact strength when compared to the other composites with related sedge fibers.

**Figure 5:**
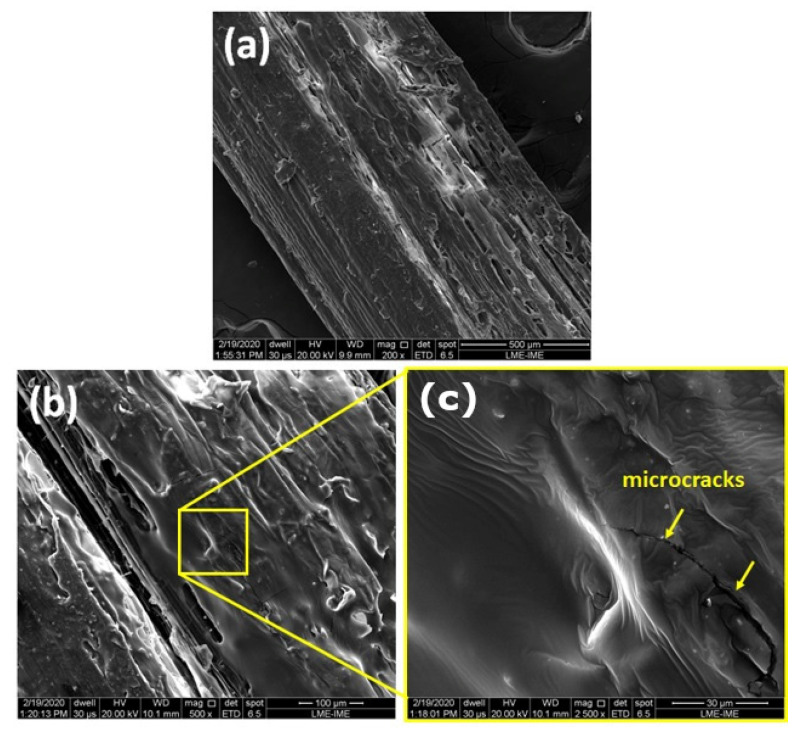
SEM images of the CM sedge fibers with magnifications of (**a**) 200×; (**b**) 500×; (**c**) 2500×.

SEM observations of the surface aspects of the fibers are shown in Figure 5. A relatively rough fiber surface can be noticed (Figure 5a), which is generally considered a good feature for composite materials, since it might improve the adhesion between fiber and matrix by mechanical interlocking in the fiber-matrix interface. However, higher magnification images showed inhomogeneities, such as the rougher regions contrasting to the smoother regions in Figure 5b. Microcracks can be observed (arrows) in the inset of Figure 5b, which might be attributed to degradation by the SEM electron beam power.

Table 5 shows the average results for the tensile strength of the CM sedge fiber-reinforced composites. Table 5 also shows literature values for the tensile strength of the same neat epoxy resin [57]. The results displayed comparatively poor tensile properties for the CM sedge-reinforced composites. In fact, the values of tensile strength for all composites were lower than that for the neat epoxy resin. This indicated that the fibers did not act as reinforcement when tensile loads were applied to the material. In this case, the fibers probably acted as defects in the material’s structure, and the tensile properties of the fiber-matrix interface were impaired. This was consistent with the small 2923 cm^−1^ band found in the FT-IR spectrum in Figure 2 for the CM sedge fiber.

**Figure 6:**
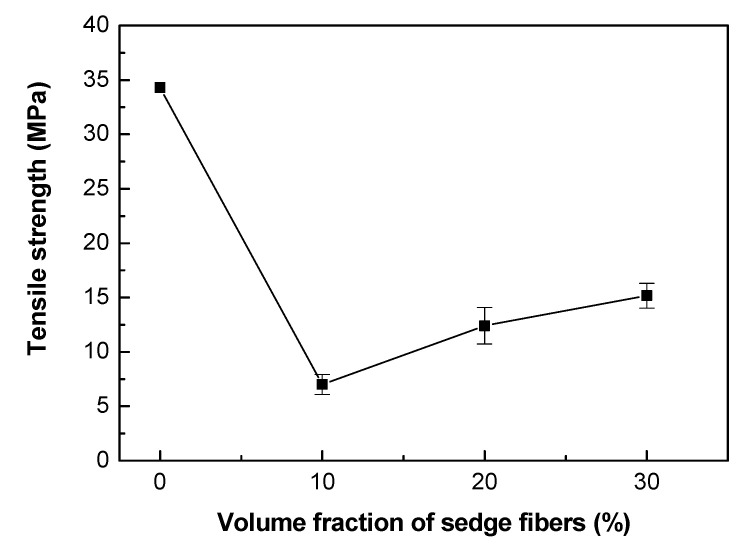
Tensile strength as a function of the fiber fraction for the neat epoxy resin and CM sedge fiber-reinforced composites.

The results in Table 5 can be better visualized by the graph in Figure 6. Although there is a tendency of increasing tensile strength with the fiber fraction, as already mentioned, the values were significantly lower when compared to the neat epoxy resin. An apparent contradiction is a fact that the sedge fibers improve the impact properties of the material. However, as observed before [56], the low interface strength resulted in a greater fracture surface. Consequently, it might provide better impact properties to the material, even though the tensile strength was lower.

Table 6 shows the ANOVA for the tensile strength of the composites. According to Table 6, the hypothesis that the averages are equal was rejected, with a level of significance of 5%, since the calculated F was higher than the critical F_c_ (tabulated). Therefore, it was confirmed that there was a significant difference between the tensile strength for the different CM sedge fiber composites.

Table 7 shows the results of the Tukey’s honestly significant difference (HSD) test for the tensile strength. The calculated HSD was 5.46 MPa, and thus the differences above the HSD were considered significant. These values are marked in bold in Table 7 and showed that the tensile strength of the 30 vol.% CM sedge fiber composites was better than all other tested specimens.

**Figure 7:**
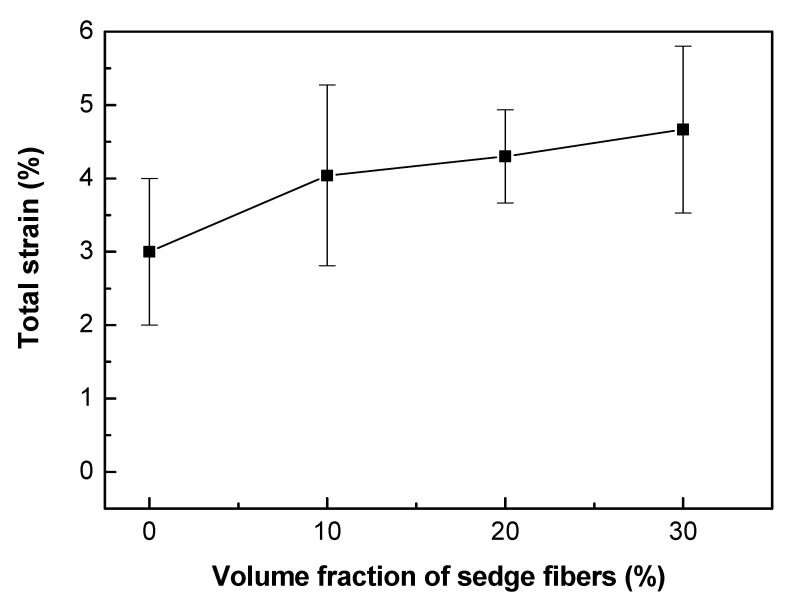
Total strain as a function of the fiber fraction for the neat epoxy resin and CM sedge fiber-reinforced composites.

**Figure 8:**
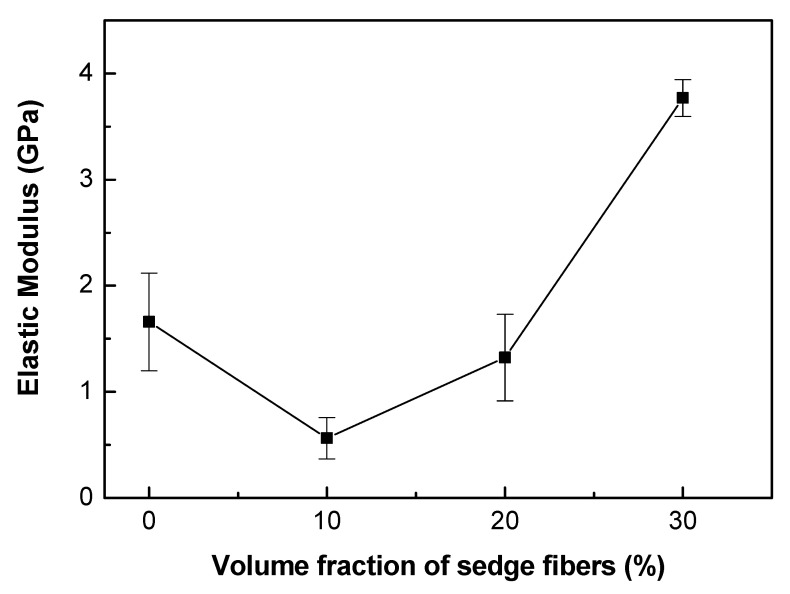
Elastic modulus as a function of the fiber fraction for the neat epoxy resin and CM sedge fiber-reinforced composites.

Furthermore, through the data obtained by the tensile test, it was possible to calculate the elastic modulus of the composites and their total strain. The results are presented in Table 8 and Figure 7 and Figure 8.

According to Table 8 and Figure 8, it is possible to observe a clear tendency of increasing the elastic modulus of the composites as the volumetric fraction of fibers increases, probably because of the higher stiffness of the fibers. Comparing to literature data for the same epoxy resin [58], only the 30 vol.% CM fiber-reinforced composite showed higher modulus, probably due to difficulties in load transfer in the fiber-matrix interface. On the other hand, the total strain graph, Figure 7, did not show any significant variation between the different materials, within the standard deviation.

Preliminary tensile results for the CM sedge fiber indicated a maximum strength of 164 MPa, an elastic modulus of 4.4 GPa, and a total strain of 4.1%. In particular, the CM sedge fiber maximum strength was significantly higher than the epoxy matrix, 34.3 MPa, in Table 5. However, the aforementioned low fiber/epoxy interfacial strength was responsible for the inferior strength of the composites in Figure 6, in spite of the higher impact energy, Figure 4, and elastic modulus, Figure 8, reinforcement effect. CM sedge fiber treatment, especially as has been done for other NFLs with graphene oxide [59], is expected to substantially improve the composites’ tensile strength.

The tensile test results were also compared to the results for the other fibers of the *Cyperus* family (Table 9), investigated by [41,43]. It is important to mention that these authors performed the tests according to the ASTM D638 standard [60]; besides, the polymeric matrices are also different, so the results might not be directly comparable to the present results. However, comparing the properties, it is worth to have some idea of the property values for similar materials. According to Table 9, the *Cyperus malaccensis* composites presented similar mechanical properties when compared to those of *Cyperus esculentus* and *C. pangorei* composites.

Table 10 shows the results of the ballistic tests. Values of absorbed energy during the impact of the projectile with the materials (E_abs_) and the calculated limit velocity (V_L_) are presented. All the specimens were perforated during the tests, so their residual velocities could be successfully measured. It is expected that the higher the absorbed energy, the better is the ballistic performance. From the data obtained, it was not shown any significant variation between the different materials.

Table 11 presents the ANOVA for the absorbed energy in the ballistic test. According to Table 11, since F calculated was smaller than F_c_ (tabulated), the hypothesis that the averages are equal was accepted (with a level of significance of 5%). Therefore, it was confirmed that there was no significant difference between the absorbed energy of the different materials.

**Figure 9:**
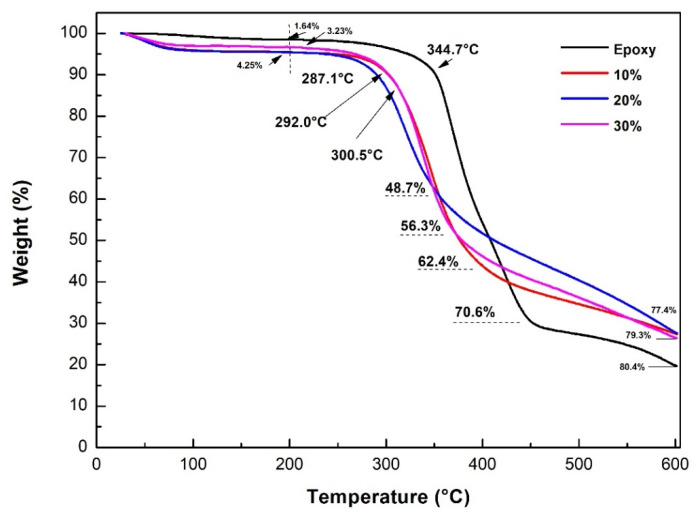
TG curves for the epoxy resin and for the CM sedge fiber-reinforced composites.

**Figure 10:**
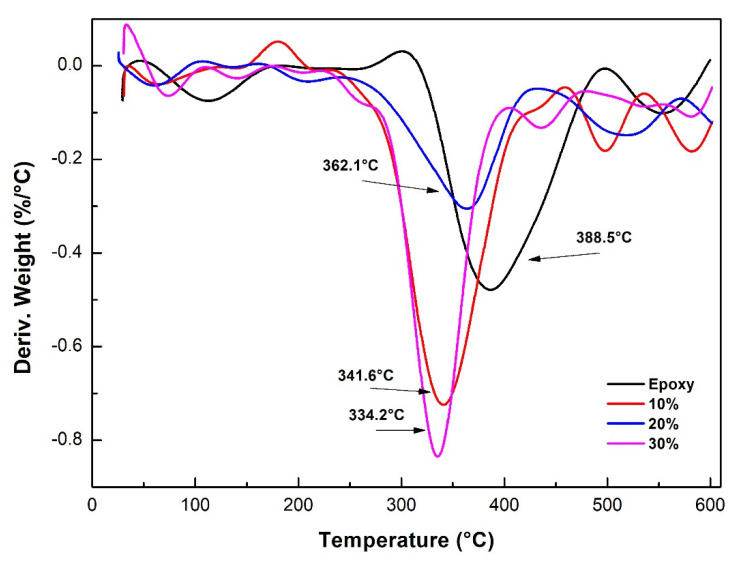
DTG curves for the epoxy resin and for the sedge fiber-reinforced composites.

Figure 9 and Figure 10 show the thermogravimetric (TG) and its derivative (DTG) curves, respectively, for the neat epoxy resin and the composites reinforced with 10, 20, and 30 vol.% of CM sedge fibers. The curves for the different materials presented similar features. Observing the TG curves in Figure 9, there was a small mass loss (<5%) at low temperatures (until 200 °C), which was attributed to moisture desorption. For the neat epoxy resin, for example, the mass loss was extremely low, 1.64%, due to its hydrophobic nature (little water absorption). For the composites, the mass loss in this stage was slightly higher, ranging from 3 to 4.2%, due to the higher moisture absorption capacity of the sedge fibers that are now present in the structure.

At higher temperatures, degradation and rupture of the polymeric chains occurred, with severe mass loss. This occurred at T_onset_ = 345 °C for the neat epoxy resin, with a maximum rate at 389 °C (Figure 10), resulting in a 70.59% mass loss in Figure 9. A third stage occurring at temperatures ranging from 455 to 600 °C resulted in a final formation of ash corresponding to about 19.6% of the initial mass, also shown in Figure 9. For the composites, the degradation occurred at lower temperatures, as previously reported [39,61], at T_onset_ = 292 °C for the 10 vol.% fiber-composites, T_onset_ = 287 °C for the 20 vol.% fiber-composites, and T_onset_ = 301 °C for the 30 vol.% CM fiber-composites. These temperatures could be considered as a first limit for the thermal stability of the CM sedge fiber-reinforced composites. Another difference between the epoxy resin and the composites was related to the temperature corresponding to the maximum rate of degradation, shown in the DTG curves in Figure 10, where the composites had a temperature ranging from 334 to 362 °C, which was slightly lower than 389 °C registered for the neat epoxy resin.

Table 12 presents the main thermogravimetric parameters, temperatures, and mass loss for the neat epoxy, 0% fiber, and CM sedge fiber-reinforced composites.

Lignin is the first structural component of NLFs to begin its degradation process at lower temperatures from 220 °C and continues above 440 °C [39]. Thus, the degradation of lignin is directly responsible for the thermal stability limit of a composite reinforced with NLFs.

**Figure 11:**
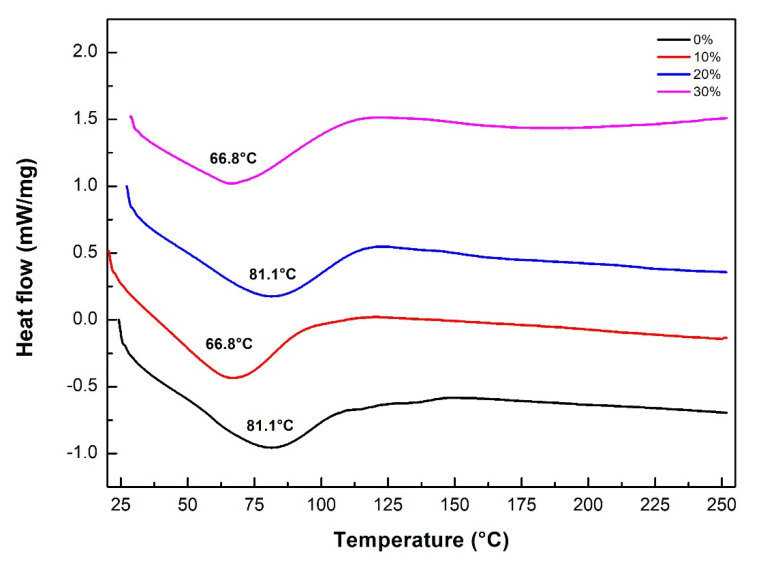
DSC curves for the neat epoxy resin and for the CM sedge fibers reinforced composites.

**Figure 12:**
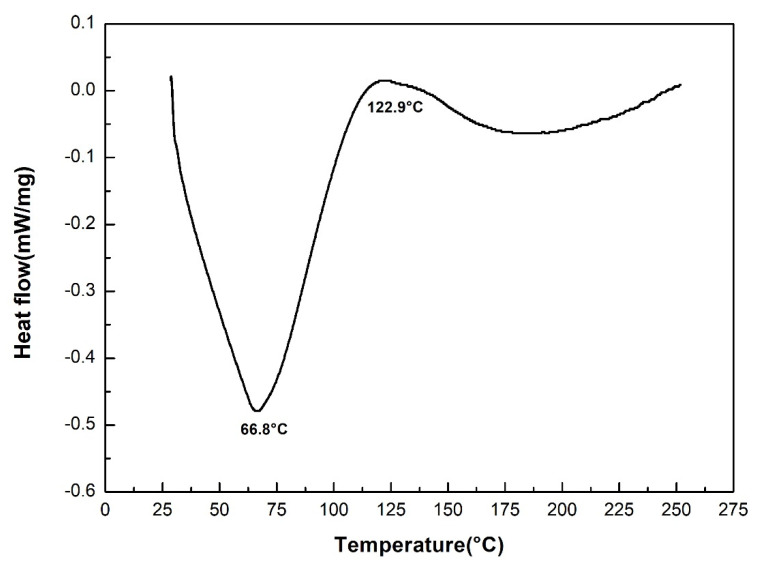
Isolated DSC curve for the 30 vol.% sedge fiber-reinforced composites.

Figure 11 shows the DSC curves for the neat epoxy resin and for the sedge fibers-reinforced composites. The curve for the epoxy resin showed an endothermic peak at 81.1 °C, which was associated with the glass transition temperature (Tg). Regarding the curves representing the composites, endothermic peaks ranging between 66.8 and 81.1 °C could be observed, also associated with the Tg. Changes in the baseline on temperatures ranging from 119 to 122.9 °C might be related to a post-curing process, as evidenced in the isolated DSC curve for the 30 vol% CM sedge fiber composite in Figure 12 [62,63]. The decrease in Tg for the composites, as compared to the neat epoxy, was due to the interference of CM sedge fiber with the 3D organization of the polymer macromolecules. This is a common event for NLF composites [39], which does not compromise the thermal stability for the usual engineering application since stiffness and hardness are not significantly changed.

## 4. Summary and Conclusions

In the present work, mechanical, ballistic, and thermal behavior of the *Cyperus malaccensis* (CM) sedge fiber-reinforced composites were, for the first time, investigated. The main conclusions are:

- There was a tendency of increasing impact strength of the CM sedge fiber-reinforced composites with increasing fiber fraction. The best result was obtained by incorporating 30 vol.% CM fibers as reinforcement, producing a 181% increase in the Izod impact energy when compared to the values obtained for the neat epoxy resin.
- The CM sedge fiber-reinforced composites presented relatively low tensile strength, showing values below that for the neat epoxy resin. This indicated that the fibers probably acted more as defects in the structure of the weak fiber-matrix interface.
- There was also a tendency of increasing elastic modulus of the composites as the CM fiber fraction increases, which was attributed to the higher stiffness of the fibers. However, a comparison to literature values for the neat epoxy resin showed that only the 30 vol.% CM sedge fiber-reinforced composite showed higher modulus than the resin. This was attributed to difficulties in load transfer through the weak fiber-matrix interface.
- The total strain and the absorbed energy during ballistic tests did not show any significant variation between the different CM sedge fiber composites.
- The thermal behavior of the neat epoxy resin and the CM sedge fiber composites were similar, showing slight differences in the temperatures in which the thermal processes occurred. First, there was little mass loss at low temperatures up to 200 °C, attributed to moisture desorption. This phenomenon was less significant to the neat epoxy resin (only 1.64% of mass loss) than for the composites (3 to 4.2% of mass loss around 100 °C). At higher temperatures, degradation and rupture of the polymeric chains occurred for all materials, with severe mass loss. This process was activated at around 345 °C for the neat epoxy resin and at lower temperatures ranging from 287 to 300 °C for the CM sedge fiber composites.
- The glass transition event was identified by the DSC method. Glass transition temperatures (Tg) were obtained as 81.1 °C for the neat epoxy resin and ranging from 66.8 to 81.1 °C for the CM sedge fiber composites.
- Both mechanical reinforcement and relative thermal stability up to 200 °C disclosed a potential application of CM sedge fiber epoxy composites as substitutes for fiberglass common use.

## Figures and Tables

**Table 1:** Izod impact energies for the neat epoxy resin and *Cyperus malaccensis* (CM) fiber-reinforced composites.

Izod Impact Energy (J/m)				
Specimen	Neat Epoxy Resin	10 vol.% Fibers	20 vol.% Fibers	30 vol.% Fibers
**1**	19.5	9.7	48.5	68.4
**2**	14.5	33.5	66.3	80.6
**3**	19.5	17.5	43.5	58.8
**4**	14.7	11.5	59.1	78.7
**5**	14.7	20.4	40.5	67.6
**6**	24.6	46.5	33.5	59.2
**7**	19.6	39.2	42.6	38.7
**8**	19.6	17.5	24.4	79.0
**9**	38.9	22.4	45.1	59.3
**10**	43.8	22.9	65.6	39.6
**Average**	**22.4 ± 9.2**	**23.3 ± 10.5**	**43.7 ± 12.3**	**63.0 ± 14.3**

**Table 2:** Analysis of variance for the Izod impact energy of the neat epoxy resin and CM sedge fiber-reinforced composites.

Causes of Variation	Degrees of Freedom	Sum of Squares	Mean Square	F (Calculated)	F_c_ (Tabulated)
Treatments	3	11188.5	3729.5	22.68	2.87
Residue	36	5918.6	164.4		
Total	39	17107.2			

**Table 3:** HSD (honestly significant difference) test for the Izod impact energy of the neat epoxy resin and CM sedge fiber-reinforced composites.

Volume Fraction of Sedge Fibers	0%	10%	20%	30%
0%	0	1.20	**23.99**	**40.09**
10%	1.2	0	**22.79**	**38.89**
20%	**23.99**	**22.79**	0	**16.1**
30%	**40.09**	**38.89**	**16.10**	0

**Table 4:** Impact resistance comparison of composites reinforced with different natural fibers belonging to the *Cyperus* family.

Matrix	Fiber Species	Volume Fraction (%)	Impact Resistance (J/m)	Reference
Epoxy	*Cyperus malaccensis*	10	23.3	PW^1^
		20	45	
		30	63	
Polypropylene	*Cyperus esculentus*	10	10	[41]
		20	18	
		30	25	
Polyester	*Cyperus pangorei*	10	4.2–7.8	[43]
		20	4.2–7.2	
		30	5.2–10.8	

^1^ PW—Present work.

**Table 5:** Tensile strength results for the CM fiber-reinforced composites.

Volume Fraction (%)	Tensile Strength (MPa)	Reference
0	34.3 ± 4.2	[57]
10	7.0 ± 3.1	
20	12.4 ± 5.5	PW^1^
30	15.2 ± 3.6	

^1^ PW—present work.

**Table 6:** Analysis of variance for the tensile strength of the CM sedge fiber-reinforced composites.

Causes of Variation	Degrees of Freedom	Sum of Squares	Mean Square	F (Calculated)	F_c_ (Tabulated)
Treatments	2	379.2	189.6	9.38	3.32
Residue	30	606.7	20.2		
Total	32	985.8			

**Table 7:** HSD test for the tensile strength of the CM sedge fiber-reinforced composites.

Volume Fraction of Sedge Fibers	10%	20%	30%
10%	0	5.4	**8.16**
20%	5.4	0	2.76
30%	**8.16**	2.76	0

**Table 8:** Total strain and elastic modulus results for the CM sedge fiber-reinforced composites.

Volume Fraction (%)	Total Strain (%)	Elastic Modulus (GPa)	Reference
0	3.0 ± 1.0	1.66 ± 0.46	[45]
10	4.0 ± 1.2	0.56 ± 0.19	PW^1^
20	4.3 ± 0.6	1.32 ± 0.40	
30	4.7 ± 1.1	3.77 ± 0.05	

^1^PW—present work.

**Table 9:** Tensile strength and elastic modulus comparison of composites reinforced with different natural fibers belonging to the *Cyperus* family.

Matrix	Fiber Species	Volume Fraction (%)	Tensile Strength (MPa)	Tensile Modulus (GPa)	Reference
Epoxy	*Cyperus malaccenis*	10	7	0.6	PW^1^
		20	12	1.3	
		30	15	3.8	
Polypropylene	*Cyperus esculentus*	10	35	1.2	[41]
		20	40	1.6	
		30	50	1.9	
Polyester	*Cyperus pangorei*	10	20–25	2–2.3	[45]
		20	20–30	2.3–2.6	
		30	30–40	2.6–2.7	

^1^PW—present work.

**Table 10:** Absorbed energy in the ballistic test and limit velocity for the epoxy resin and for the CM sedge fiber-reinforced composites.

Absorbed Energy (J)			
Performed Shootings	10 vol.% Fibers	20 vol.% Fibers	30 vol.% Fibers
**1**	82.5	83.2	74.9
**2**	81.7	94.7	93.0
**3**	86.0	71.0	72.7
**4**	77.9	62.0	60.2
**5**	74.5	70.8	69.0
**Average value**	**80.5 ± 1.5**	**76.3 ± 2.5**	**74.0 ± 2.5**
**Limit velocity (m/s)**			
**Average value**	**221.5 ± 5.5**	**215.2 ± 15.9**	**212.5 ± 15.2**

**Table 11:** Analysis of variance for the absorbed energy in the ballistic test of the CM sedge fiber-reinforced composites.

Causes of Variation	Degrees of Freedom	Sum of Squares	Mean Square	F (Calculated)	F_c_ (Tabulated)
Treatments	2	109.0	54.5	0.50	2.81
Residue	12	1306.4	108.9		
Total	14	1415.5			

**Table 12:** Thermogravimetric parameters for the neat epoxy and the CM sedge fiber-reinforced composite.

Volume Fraction of Sedge Fiber (%)	Mass Loss up to 200 °C (%)	Initial Degradation Temperature (°C)	Temperature of Maximum Degradation Rate (°C)	Mass Loss at the End of Second Stage (%)	Mass Loss at 600 °C (%)
0	1.64	344.7	388.5	70.6	80.4
10	4.23	300.5	341.6	62.4	79.3
20	4.18	287.1	362.1	48.7	77.6
30	3.23	292.0	334.2	56.3	77.4
